# Open data, open curation

**DOI:** 10.1038/sdata.2018.204

**Published:** 2018-09-25

*Scientific Data* has released a series of templates designed to help authors further engage with, and enhance, our metadata creation process.

After being accepted for publication, each of *Scientific Data*’s Data Descriptors undergoes a curation process. During this process our in-house curators work with the authors to check that the associated data records are accurately cited, and that the provenance of the resulting data files is clear.

The most tangible output of the curation process is the machine-accessible metadata record that forms a part of each Data Descriptor (in ISA-Tab format^1^; http://isa-tools.org). This serves as a record of the curation process, and as a standardized account of the key experimental steps that led to generation of the data. The records are used to create a metadata summary table, which appears after the abstract of each Data Descriptor, allowing readers to understand key study characteristics at-a-glance. The metadata records also power ISA-explorer, an experimental data discovery tool that supplements the journal and provides a user friendly interface to view the full machine-accessible record (http://scientificdata.isa-explorer.org)^2^. The metadata records are shared under a CC0 waiver (https://creativecommons.org/share-your-work/public-domain/cc0) and can also be downloaded directly from each Data Descriptor.

Curation has other less visible, but equally valuable benefits. For example, creating a metadata record requires mapping each study ‘input’ to a specific data file ‘output’. This process of input/output accounting can help identify errors that may have been overlooked during peer review and which may impact data reusability.

The majority of identified issues are minor. For example, a manuscript might state 36 samples were generated, whereas the data files show the presence of 38 samples, or our curators may advise authors to edit their prose to clarify sample or data provenance. In some cases, however, more serious issues may be found. For example, our curators have come across instances where the wrong data files have been uploaded to the data repository, or where important discrepancies existed between the description of the data and the actual files at the repository.

Having identified potential issues, our curators work closely with our authors to edit the manuscript or data archive files as required, to ensure that the final published Data Descriptor and data archive maximally facilitate reuse of the generated data. In more serious cases, we may return to our Editorial Board or referees for guidance. This process means that all identified errors (minor or major) are remedied prior to publication, resulting in final publication of data that are easier to reuse.

To ensure preservation of data access over the longer term, each of the formal data citations in our publications is checked for formatting and accuracy by our curators. For repositories that assign accession identifiers (IDs), we check the accuracy of the accession ID, the corresponding repository name, and check that these refer to the relevant data files in the most direct way possible. For datasets with digital object identifiers (DOIs), our curators check that the repository name is consistent with that registered at DataCite for each citation^3^.

To record data provenance, our curators refer to the accepted manuscript to establish which method(s) resulted in the generation of each data file. Data file integrity is checked at this stage, with our curators spot-checking the data files to ensure that they are downloadable. The data file archiving structure is checked against the descriptions provided in the manuscript and in any metadata files at the repository. The licence information at the repository is also checked to ensure it matches that declared in the Data Descriptor.

Our authors are a vital part of the curation process, and it is important that they should feel an ownership of the generated metadata records, since these records will form part of their final publication. In addition, we firmly believe the best-placed people to check the accuracy of the recorded experimental steps, are those who generated the data in the first place, i.e. our authors. We appreciate, however, that generation of detailed metadata can be initially daunting. Researchers who have published with us in the last year may be aware that we have been piloting a set of metadata templates designed to make it easier for our authors to participate more actively in the curation process.

During this pilot phase, we have been refining both the templates and our workflow in response to the feedback collected. While capturing data generation workflows in a structured format may be unfamiliar to many researchers, we found most authors taking part in the pilot were able to complete the provided templates easily. This facilitated authors’ contributions to the curation process, and also reduced the time to publication by helping authors to self-identify potential discrepancies earlier in the publication process.

We are therefore pleased to announce the roll-out of our metadata templates for use by all of our authors. Templates for a selection of different disciplines and data-types are available from our author guidance pages (https://go.nature.com/sdatametadata). Authors are invited to include a completed metadata template with their initial submission. If not included at initial submission, the editorial office will request completion of the relevant metadata template during revision, when appropriate.

We feel that our curation process forms a natural complement to traditional peer-review. By helping our authors engage with this process earlier and more directly, we hope to further reduce the time taken to publish our authors’ work, whilst simultaneously helping our authors to increase the value and reusability of the data they have generated. Promoting metadata generation as a regular part of publishing a Data Descriptor aligns with our wider goal of helping researchers to not just share data, but to share data well.
